# Index Medicus

**Published:** 1879-01-20

**Authors:** 


### *INDEX MEDICUS.*

Dr. Jno. S. Billings, Surgeon U. S. Army, and Dr. Robert Fletcher, M. R. C. S. Eng., have commenced the publication of a monthly classified Record of the current medical literature of the world. The titles of valuable original articles in medical journals and society transactions will be given, and the places where found, etc. Also description of new books, with size, price and illustrations.

